# Natural resistance to cancers: a Darwinian hypothesis to explain Peto’s paradox

**DOI:** 10.1186/1471-2407-12-387

**Published:** 2012-09-03

**Authors:** Benjamin Roche, Michael E Hochberg, Aleah F Caulin, Carlo C Maley, Robert A Gatenby, Dorothée Misse, Frédéric Thomas

**Affiliations:** 1 IRD, UMMISCO (UMI IRD/UPMC), 32, avenue Henry Varagnat, 93143, Bondy Cedex, France; 2 ISEM, Institut des Sciences de l’Evolution, Université Montpellier 2, CNRS, Case Postal 65, Place Eugène Bataillon, 34095, Montpellier cedex 5, France; 3 Santa Fe Institute, 1399 Hyde Park Road, Santa Fe, NM, 87501, USA; 4 Genomics and Computational Biology Graduate Program, University of Pennsylvania, Philadelphia, PA, 19104, USA; 5 Center for Evolution and Cancer, Helen Diller Family Comprehensive Cancer Center and Department of Surgery, University of California San Francisco, San Francisco, CA, 94143, USA; 6 Departments of Radiology and Integrated Mathematical Oncology, the Moffitt Cancer Center, 12902 Magnolia Dr, Tampa, FL, 33612, USA; 7 IRD, MIVEGEC (UMR CNRS/IRD/UM1), 911 Ave. Agropolis, BP 64501, FR-34394, Montpellier cedex 5, France; 8 CREEC, Universite Montpellier 2, CC, 06003, 95 rue de la Galera,, 34095, Montpellier Cedex 5,, France

## Abstract

**Background:**

Peto's paradox stipulates that there is no association between body mass (a surrogate of number of cells and longevity) and cancer prevalence in wildlife species. Resolving this paradox is a very promising research direction to understand mechanisms of cancer resistance. As of present, research has been focused on the consequences of these evolutionary pressures rather than of their causes.

**Discussion:**

Here, we argue that evolution through natural selection may have shaped mechanisms of cancer resistance in wildlife species and that this can result in a threshold in body mass above which oncogenic and tumor suppressive mechanisms should be increasingly purified and positively selected, respectively.

**Summary:**

We conclude that assessing wildlife species in their natural ecosystems, especially through theoretical modeling, is the most promising way to understand how evolutionary processes can favor one or the other pathway. This will provide important insights into mechanisms of cancer resistance.

## Background

**Figure 1:**
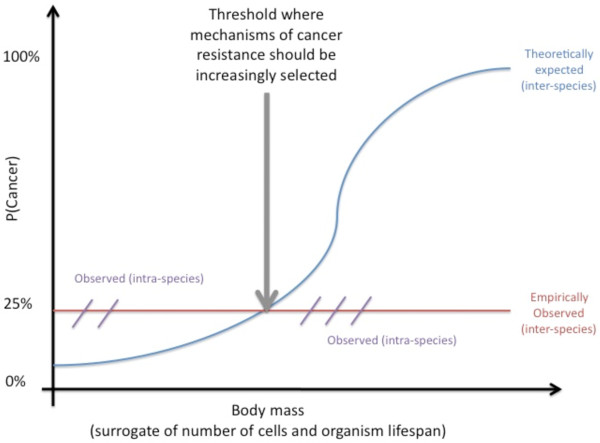
**Summary of the current knowledge on Peto’s paradox according to the spare data available reviewed in [**[2]**].** Body mass seems to be a relevant surrogate for cancer prevalence at an intra-species level, but not an inter-species level.

One of the challenges to multicellular organisms is the deregulation of the cooperative interactions between component cells that natural selection has taken long periods to produce. Cancer is one large class of such multicellular deregulation often resulting in death [1], and poses the puzzle of how or whether it is opposed by natural selection. Although assessing natural selection in human populations is challenging, indications of how natural selection may have moulded human cancer incidence can be assessed by comparing incidences of cancers in wildlife species [2]. Specifically, we need to understand the mechanisms underlying the so-called “Peto’s paradox”—the absence of a correlation across species between cancer and body size or longevity [2,3], Figure 1. If each dividing cell in a multicellular organism has the same probability of initiating a malignant neoplasm, then all else being equal, the more cells an organism has, the greater the chance of cancer emerging. Thus, blue whales which, at adulthood, may weigh more than 100 metric tons, should be at least 1000 times more likely to develop a cancer than humans. Moreover, transitions to malignancy are expected to increase with the number of cell divisions—that is with organism life-span. Numerous studies have shown correlations between longevity and body size, making Peto’s paradox all the more difficult to resolve [2]. How do large, long-lived species overcome the burden of cancer? We [2] hypothesized that organisms evolving large body sizes and long life spans, concomitantly evolved mechanisms to offset increased cancer risk. These may include slower somatic cell turnover, redundancy of tumor suppressor genes, more efficient immune systems, better suppression of inflammation or resistance to oncogenic viruses. Currently, no clear consensus has emerged about which of these mechanisms, if any, are the products of natural selection. It is therefore crucial to focus more attention on the evolutionary causes of Peto’s paradox.

## Discussion

Here, we propose that oncogenic or tumor suppressive mechanisms should be increasingly purified or positively selected, respectively, above a given threshold in body mass (Figure 1). There is correlative evidence for a within-species increase in cancer frequency with body mass [4]. For instance, cohort studies in humans have shown that leg lengths 3-4 mm above the average is associated with a 80% higher risk of non-smoking cancers (Figure 1; reference 4, but see 5). Despite the prediction that more cells in a body should be associated with a higher probability of cancer, current knowledge indicates that cancers do not increase across wildlife species as a function of body mass (incidences are typically between 20 and 40%, 2).

We suggest that these contrasting patterns are produced by large wildlife species undergoing stronger selection against cell deregulation and for tumor suppression than smaller ones. Analyzing the selective pressures within a species’ ecosystem (i.e.*,* biotic and a biotic environments) will inform if natural selection targets oncogenic and/or tumor suppressive mechanisms. Identifying patterns across species in cancer resistance evolution would be an important insight into resolving Peto’s paradox.

We need to understand the selective (biotic and a biotic) landscapes in which species evolved and continue to evolve. Indeed, natural selection is the product of how environments favor specific heritable phenotypes. Cancer vulnerability amongst wildlife species is likely to have been shaped by natural selection, depending on which fitness-reducing risks predominate (somatic diseases including cancer, infectious and parasitic diseases, predation and adverse environmental conditions). For instance, small rodents *in natura* may succumb to cancer, but only if they do not first die from any one of numerous other causes, such as predators, infectious diseases, or environmental vagaries such as floods, temperature extremes, etc. Natural selection will tend to promote resistance to sources of mortality prior to reproduction, meaning that for blue whales to grow so large and live so long, they need to both develop defenses against predators and resistance to somatic diseases like cancer. There is clearly a chicken-and-egg problem here, since changes in body size, longevity and life history strategy will alter the selective influence of different mortality factors, including the probability of cancer emergence.

**Figure 2:**
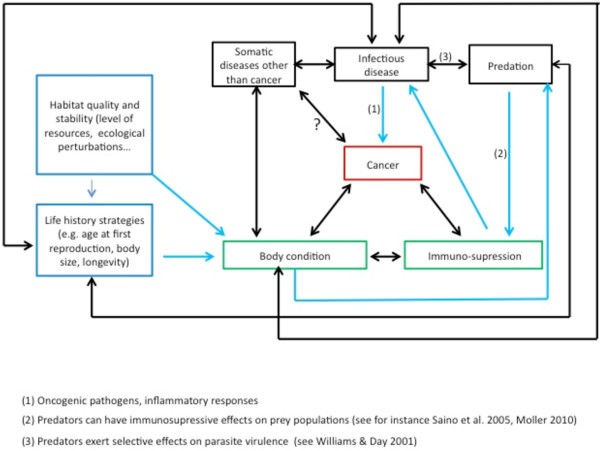
**The network of interactions between cancer and various variables acting on individuals and species in ecosystems.** Arrows represent direct (e.g. *,* oncogenic pathogens cause cancer) or evolutionary (e.g. *,* predation exert selective pressure on parasite virulence) responses. Arrows are in two colors for clarity (blue for unidirectional relationships, dark for reciprocal, or potentially reciprocal, relationships).

Studying this complexity (i.e.*,* numerous environmental factors acting and interacting in opposite directions and/or with reciprocal effects (Figure 2)) requires the development of a theoretical approach. Adopting a quantitative framework, such as adaptive dynamics [5], widely applied to understanding the evolution of pathogens and life-history traits, can help understand how different biotic and a biotic selective pressures affect trait evolution and especially those involved in cancer protection.

Since wildlife species are subjected to a large variety of selective pressures and are found in a diverse range of habitats, it should be possible to use comparative genomics [6] to understand how proto-oncogenes and TSGs covary with certain environmental characteristics. Indeed, comparing genomic regions of interest for cancer research, e.g.*,* proto-oncogenes or Tumor Suppressor Genes widespread in mammals, according to the biotic and a biotic environments where these species are found can give important insights into how these classes of genes have been shaped by natural selection as a function of the environment.

Addressing these considerations is undoubtedly relevant for human populations living in different environmental conditions (e.g.*,* presence or absence of pathogens). Indeed, evidence suggests that many human populations lack alleles with enhanced protection against certain cancers, possibly because their short life-spans have precluded selection for those alleles [4,7,8].

## Summary

Given the complex nature of interactions arbitrating cancer (Figure 2), mathematical and statistical approaches will be necessary to tease apart causal mechanisms and their interactions [9-19], especially for the optimal use of chemotherapies [20]. In particular, quantitative approaches will help understand the selective forces acting on oncogene dynamics in animals in nature. Although cancer is an important cause of morbidity and mortality in several wildlife species [21], data are difficult to collect because most wild animals live and die unseen [21]. In this context, quantitative approaches will be of considerable interest to understand and predict wildlife cancers, and to see to what extent insights can instruct on the prevention of cancer in humans.

## Competing interests

The authors declare that they have no competing interests.

## Authors’ contributions

BR, MEH and FT conceived the study, BR, MEH, AFC, CCM RAG, DM, and FT wrote the manuscript. All authors read and approved the final manuscript.

## Pre-publication history

The pre-publication history for this paper can be accessed here:

http://www.biomedcentral.com/1471-2407/12/387/prepub
